# Reliability of a Portable Nonfixed Stereophotogrammetry System for Facial Imaging

**DOI:** 10.1093/asjof/ojag078

**Published:** 2026-08-06

**Authors:** Marcelo Germani, Ana C N Carnevali, Pietra Roschel de Borba, Gabriela Giro, Victor R M Munoz-Lora

## Abstract

Stereophotogrammetry (3D-SQ) provides a precise alternative for the assessment of facial aesthetic procedures. Nevertheless, its accuracy could be influenced by the operator when a nonfixed device is used. The objective of the present observational study was to evaluate the reliability of a portable nonfixed 3D-SQ device. Three operators took two 3-dimensional (3D) image captures of 3 distinct volunteers using a portable nonfixed 3D-SQ device. The acquisition of images was performed at 2 different time points (F1 and F2) with a 60 min interval between each capture for each volunteer. No external lights or tripod were utilized, as recommended by the manufacturer. The photographs were processed through specialized software to generate 3D models, and a specific region of the face was analyzed to assess the reliability of the method. Interclass correlation coefficients (ICCs) of the area, perimeter, and volume of the selected region were calculated. The results demonstrated a high intraoperator consistency at all 3 obtained values (volume ICCs = 0.829; area ICCs = 0.828; perimeter ICCs = 0.673). However, a low reliability in all values across different operators was found (ICCs < 0.5). These results suggest that variations in the captures may differ among different operators. These results may be attributed to factors such as the operator height, differences in reference points, or equipment stability. The study concluded that the reliability in facial stereophotogrammetry using a nonfixed device can be influenced by the operator, exhibiting high intraoperator reliability but significant variations between different operators.

**Level of Evidence**: 4 (Therapeutic) 
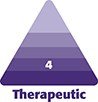

The demand for aesthetic treatments has significantly increased over the years. These treatments are effective because of their multifaceted and continuous nature, yielding predictable and long-lasting results.^1^ Usually, diagnosis and evaluation of these results are measured through 2-dimensional (2D) photographic assessments. However, this method is limited in capturing the depth and volume of structures.^2^ Moreover, several factors, such as proper external lighting and the operator's skill in configuring and handling the camera, are essential for the reliability of the images.^3^

As an alternative, facial scanning systems have been adopted for planning, outcome assessments, and research of aesthetic treatments.^4,5^ These systems may differ on the technology used to create 3-dimensional (3D) models and even the process for taking the images. Stereophotogrammetry quantification (3D-SQ), one of these technologies, computationally analyzes 2 or more simultaneous photographs to generate a 3D geometric model.^6,7^ There are 2 main types: active technology, which projects an unstructured pattern of light (visible or infrared) onto the object's surface; and passive, which relies solely on natural ambient light. 3D-SQ stands out for being portable, radiation free, and allowing rapid image capture.^7^

However, 3D-SQ is not fully automated and depends on the operator’s knowledge and ability to handle the device. This fact is particularly relevant in nonfixed portable devices where the operator may significantly influence the fidelity of the results.^4^ Therefore, this study is aiming to assess the precision and reliability of a nonfixed portable 3D-SQ device for facial analysis. The study followed the STROBE guidelines.

## METHODS

The present study received the approval from the Research Ethics Committee of the Faculty of Medicine of Marília-SP (São Paulo, Brazil), under protocol number CAAE—73023923.4.0000.5413.

### Study Design

The study included 3 volunteers randomly chosen from Guarulhos University, 2 females and 1 male, who served as models for image capture, and 3 different operators. All operators underwent detailed training before starting the evaluation to ensure standardization and accuracy in image capture. The 3D-SQ device used (LifeViz, QuantifiCare, San Francisco, CA) was configured with factory-established settings. No external lights, no tripod, or any other stabilizing support was used, adhering strictly to the manufacturer's usage recommendations.

### Image Capture

**Figure 1.**
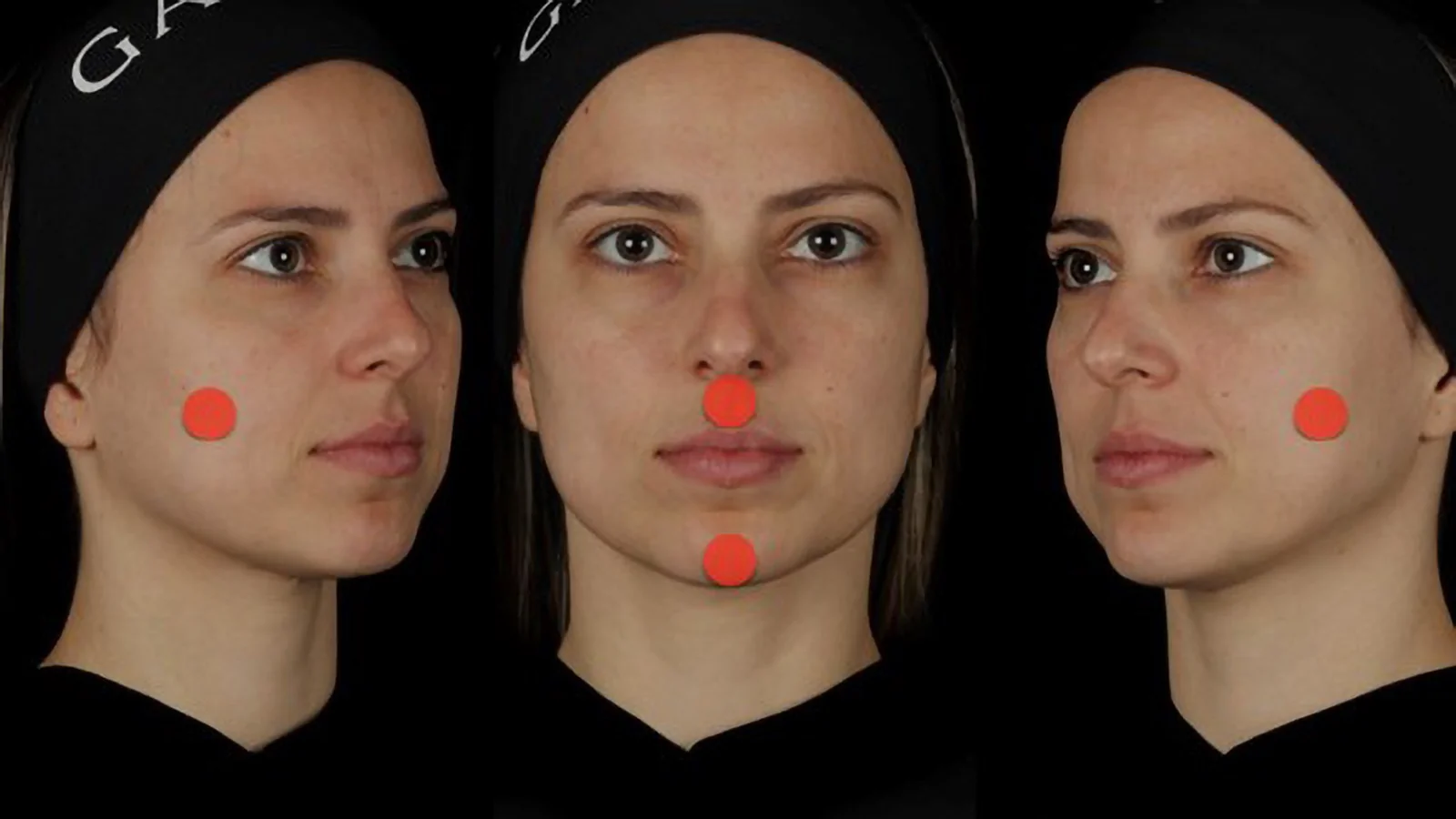
A 32-year-old female patient who underwent 3-dimensional facial analysis and the predefined anatomical points for images acquisition.

**Figure 2.**
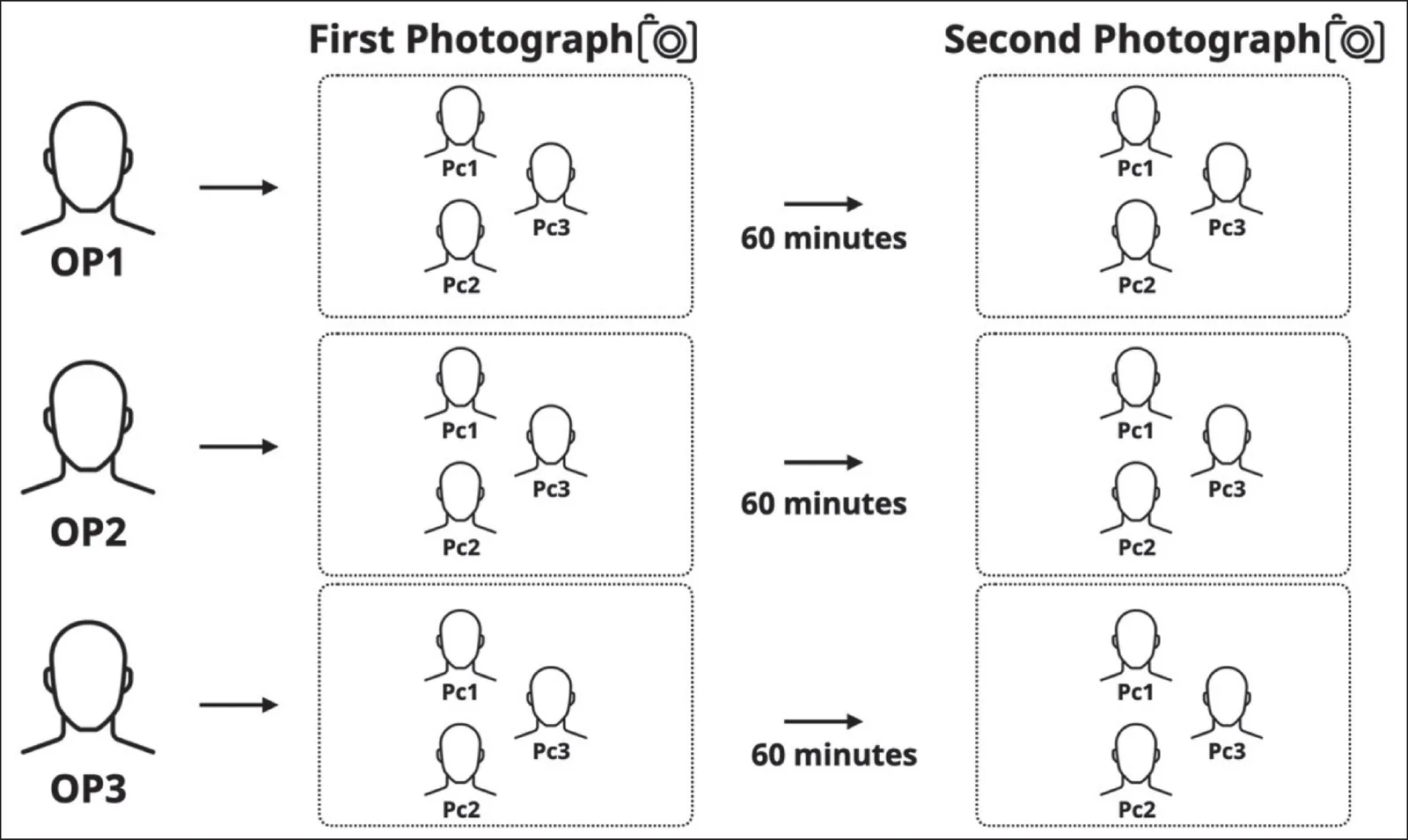
Schematic representation of the study design.

The 3D model acquisition consists of capturing a set of four 2D images of the face at predefined anatomical points: the nasal base, the left and right submalar regions, and the midline of the chin, using the 3D-SQ device (Figure 1, Videos 1, 2). To ensure the accuracy and consistency of the images, 2 round red lights are projected from the device and must be precisely aligned before each 2D image is taken.^8,9^ Each operator captured two 3D models from the 3 different volunteers (F1 and F2), within a 60 min interval between each set (Figure 2).

### 3D Analysis

All sets of four 2D images were processed using specialized software (Dermapix, QuantifiCare) to obtain the 3D models. This processing was performed by a blinded and experienced professional who was not involved in image acquisitions.

**Figure 3.**
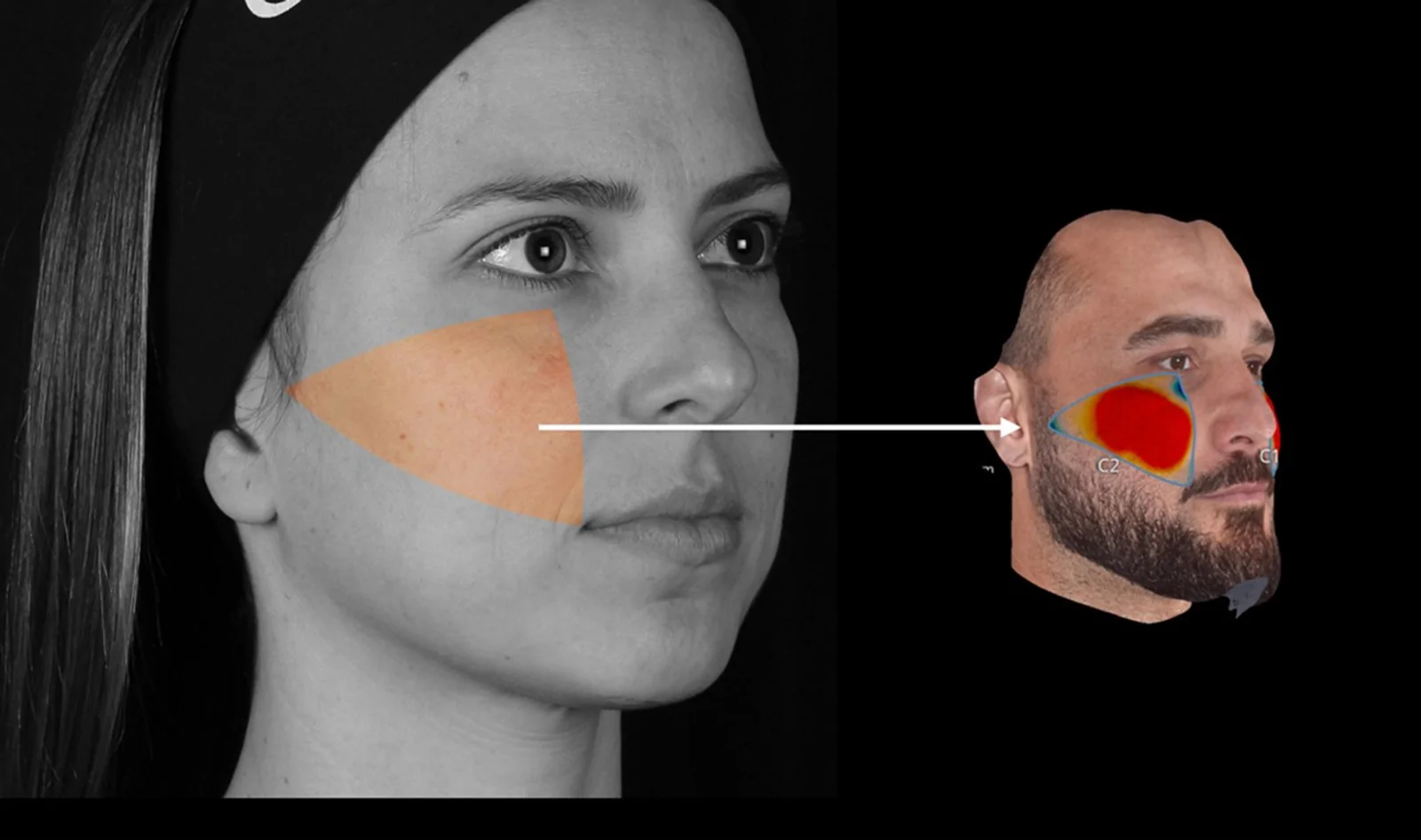
A 32-year-old female patient and a 34-year-old male patient who underwent 3-dimensional facial analysis and the delineation of the assessed facial area.

A specific facial region was selected to assess and compare the device reliability. This region was marked by a perimeter traced from a vertical line extending from the medial pupillary line to the labial commissure, then to the tragus, and returning to the medial pupillary line (Figure 3). Once manually marked, this perimeter was automatically replicated in the other 3D models of the same volunteer using a 3D-Track tool.

The 3D-Track is an automated tool integrated into the analysis software. This tool utilizes artificial intelligence (AI) algorithms to enable continuous monitoring of changes over time, minimizing the need for manual interventions. A notable aspect of the 3D-Track tool is the automatic replication of the selected perimeter of interest in the same volunteers, which ensures remarkable consistency in the measurements and significantly reduces the likelihood of human error. Then, the software provided a table containing the perimeter, area, and volume of each delineated region.

### Statistical Analysis

The accuracy of the portable stereophotogrammetry device was assessed through intraoperator and interoperator precision analyses. All possible comparisons between volunteers and operators were conducted.

Data analysis was conducted using the Jamovi statistical program (The Jamovi Project, version 1.6.23). A CI of 95% was used for statistical significance, so *P*-values below .05 were considered statistically significant. A 2-way repeated measures analysis of variance (2-way RM ANOVA) test was used to evaluate the influence of operator and time on volume, area, and perimeter.

The reliability was assessed by calculating the interclass correlation coefficients (ICCs) between test and retest outcome parameters. ICCs of volume, area, and perimeter for intra- and inter-group data were obtained. ICC values >0.75 were considered good, and values <0.5 were considered poor.

## RESULTS

In total, eighteen 3D models from 3 different volunteers were acquired from F1 (nine 3D models) and F2 (nine 3D models). In the comparisons using the 2-way RM ANOVA, no significant differences were observed in the domains of area (*P* = .374), perimeter (*P* = .314), or volume (*P* = .316) in both assessments. These results suggest a remarkable consistency in measurement data conducted by the same operator at different times and by different operators.

Moreover, the ICCs showed high levels of confidence in intraoperator analyses. All evaluated domains exhibited a high level of intraoperator consistency: volume (ICCs = 0.829; lower value 0.621 and upper value 0.975), area (ICCs = 0.828; lower value 0.402 and upper value 0.998), and perimeter (ICCs = 0.673; lower value 0.29 and upper value 0.993). On the other hand, the reliability of interoperator assessment proved to be low in all evaluated domains (ICCs < 0.5; volume = 0.433, area = 0.019, and perimeter = 0.187), as depicted in Table 1.

**Table 1.** Interclass Correlation Coefficient for Intra and Inter-group Analysis

Parameter	Intra-group analysis			Inter-group analysis		
	ICC (95% CI)	Lower	Upper	ICC (95% CI)	Lower	Upper
Volume	0.891	0.621	0.975	0.433	0.0457	0.515
Area	0.828	0.402	0.998	0.0196	0.00275	0.357
Perimeter	0.673	0.29	0.993	0.187	0.344	0.498

## DISCUSSION

According to the results of this study, high reliability can be observed in images captured by the same operator at different times (F1 vs F2). It is plausible that the high intraoperator reliability stems from the ability of each operator to consistently standardize the essential anatomical points for photographic capture. This standardization facilitates the precise replication of image conditions across multiple sessions, contributing significantly to the reliability of the collected data.^10^

Furthermore, a critical factor is the measurement and processing of images from the same volunteer using the 3D-Track tool, with the support of AI algorithms. The use of AI not only enhances the accuracy of analyses but also boosts efficiency in data processing through standardizing the marking of the area to be assessed, minimizing variations and errors that could be introduced by manual analyses. However, it is important to note that the AI tool cannot compensate for errors introduced during the initial image acquisition process, such as incorrect positioning or equipment instability, which are highly dependent on the operator.

On the other hand, the interoperator analysis demonstrated low consistency and reliability, indicating that different operators may exhibit variations of measurements. These variations can be attributed to factors such as the operator's height, distinct understanding of the anatomical reference points, and/or equipment stability during capture, which could significantly affect measurements performed by the software. Thus, it emphasizes the importance of the same operator consistently accompanying the follow-up of each patient to avoid distortions and misconceptions regarding volumetric changes or tissue displacements.

In a comparative study, Almadori et al assessed 5 different stereophotogrammetry system.^4^ It was demonstrated that the Artec Eva and Vectra H1 systems showed high reliability both intra- and inter-observer, whereas the LifeViz Mini exhibited moderate reliability. It is crucial to note that the study by Almadori et al does not specify whether stabilization methods were employed, a factor that may influence the obtained results. The difference in performance compared with other systems with higher interoperator reliability, such as the Vectra system, is likely because of the use of facial stabilizers in those studies, which eliminates the primary source of error related to patient positioning and operator stability.

Nogueira et al reported high reliability, with indices above 0.9 in another stereophotogrammetry model (Clone3D).^3^ However, it was used as a nonportable fixed device, which may favor photographic capture. On the other hand, this device is extremely large and impractical for use in diverse environments, given its fixed nature.

In a study conducted by Ceinos et al, a methodological approach distinct from that employed in our research was observed.^8^ In this previous study, the authors utilized fixed points on the face to assess intraoperator and interoperator reliability using the LifeViz system. The results indicated high intraoperator reliability for all evaluated segments, with ICCs ranging from 0.902 to 0.960. Similarly, interoperator reliability was high for most segments, with ICCs ranging from 0.739 to 0.977. As reported by the authors, these findings suggest that the LifeViz system is effective for measuring facial distances. It is important to note that, unlike our study, Ceinos and colleagues' work utilized a facial stabilizer, which may have contributed to greater interoperator reproducibility. However, it should be emphasized that, at that time, the current AI tool was not available, which could potentially influence the obtained results.

A crucial aspect to consider when assessing the reliability of stereophotogrammetry systems is the size of the analyzed area. Smaller areas may be more susceptible to measurement challenges by the software, influenced by variables such as head positioning, gaze direction, muscle tone, and facial expressions.^11^ Therefore, we chose to collect data within a reduced time interval to minimize potential variations. A systematic review published in 2022, investigating the reliability of these systems, highlighted that the periorbital region is particularly prone to measurement errors. This finding underscores the importance of considering the anatomical complexity and mobility of facial regions when evaluating the accuracy of these devices in different areas of the face.^12^

Despite a total of eighteen 3D models were done throughout the study, the small number of operators and volunteers may be considered as a limitation of this study. On the other hand, one of the strengths includes the use of a specific software tool for image measurement (3D-Track), which automatically replicates the area to be measured without manual intervention. The use of this tool ensures the standardization of all assessments conducted on patients, unlike a manual selection of the evaluated area in each 3D image, which depends on greater expertise and attention to detail by the evaluator.

## CONCLUSIONS

The study concluded that the reliability in facial stereophotogrammetry using a nonfixed device is highly influenced by the operator. Although it demonstrated high intraoperator reliability, the significant variations between different operators suggest the need for rigorous standardization and training to ensure clinical and research reproducibility. The primary clinical guidance is that the same operator must consistently perform the image acquisition for patient follow-up.
